# Endoscope-Assisted Bilateral Supraorbital Craniotomies for Reconstruction of a Traumatic Cerebrospinal Fluid Leak in a Complex Skull Base Fracture: A Case Report

**DOI:** 10.1055/a-2984-6319

**Published:** 2026-10-28

**Authors:** Toshiki Ishikura, Kentaro Horiguchi, Koichiro Maeda, Shinichi Origuchi, Yoshinori Higuchi

**Affiliations:** 1 Department of Neurosurgery 47708 Chiba University Graduate School of MedicineChibaJapan

**Keywords:** skull base, reconstruction, endoscopic surgery, cerebrospinal fluid leak, trauma, pericranial flap

## Abstract

**Background** Surgical management of traumatic cerebrospinal fluid (CSF) leakage traditionally requires extensive craniotomy. Here, we present a tailored supraorbital craniotomy using an endoscopic technique for complex skull base trauma.

**Methods** We report the case of a patient with extensive anterior skull base fractures and persistent CSF rhinorrhea despite lumbar drainage.

**Case Description** A 21-year-old woman underwent surgery for worsening CSF rhinorrhea.

**Results** Bilateral supraorbital craniotomy was performed to bypass the midline fractures and reduce the risk of venous sinus injury. Endoscopic visualization revealed a fistula associated with brain herniation. Successful multilayered reconstruction was achieved using a split pericranial flap. The patient recovered without any recurrence of CSF leakage.

**Conclusions** Combining endoscopic visualization with customized craniotomy to avoid unstable fracture lines enables safe, robust, and effective repair of CSF leaks.

## Introduction

Traumatic cerebrospinal fluid (CSF) leaks complicate approximately 12–30% of frontal skull base fractures. Although 50–80% of leaks resolve spontaneously within 1 to 3 weeks, some cases require surgical intervention. 1 Because 7–30% of CSF leaks via the nasal cavity result in meningitis, prompt and appropriate management is crucial. 2 Traditionally, traumatic skull base fractures with CSF leakage have been treated using large craniotomies. 3 Given the substantial risk of posttraumatic infection, extensive craniotomies have often been favored. 4 However, advances in endoscopic techniques have expanded potential indications for these cases. 5 Here, we report a case of skull base fracture in which endoscopic repair of CSF leak was successfully performed.

## Methods

We repaired the traumatic CSF leak using a tailored transcranial approach that avoided extensive craniotomies and endonasal endoscopic routes. Instead, a bilateral subfrontal endoscopic approach was used, enabling close visualization of the surgical site through the limited cranial openings. We evaluated the clinical feasibility of this approach and reviewed the relevant literature. This study was approved by the Institutional Review Board of Chiba University Graduate School of Medicine (approval no. HK202510–17). The requirement for written informed consent was waived, and patient consent was obtained using the opt-out method.

## Case Description

A 21-year-old woman was transported to our hospital after falling from a height. On admission, her Glasgow Coma Scale score was 8 (E1V2M5), bilateral pupils measured 5 mm, and blood pressure was 97/49 mm Hg.

After the initial resuscitation, the patient underwent surgery for pelvic fractures, hemopneumothorax, and limb fractures. Head computed tomography (CT) revealed extensive anterior skull base fractures involving the bilateral optic canals, along with frontal and temporal bone fractures ( Fig. 1A–C ). Three-dimensional (3D) CT angiography revealed no vascular injury ( Fig. 1D ).

**Fig. 1:**
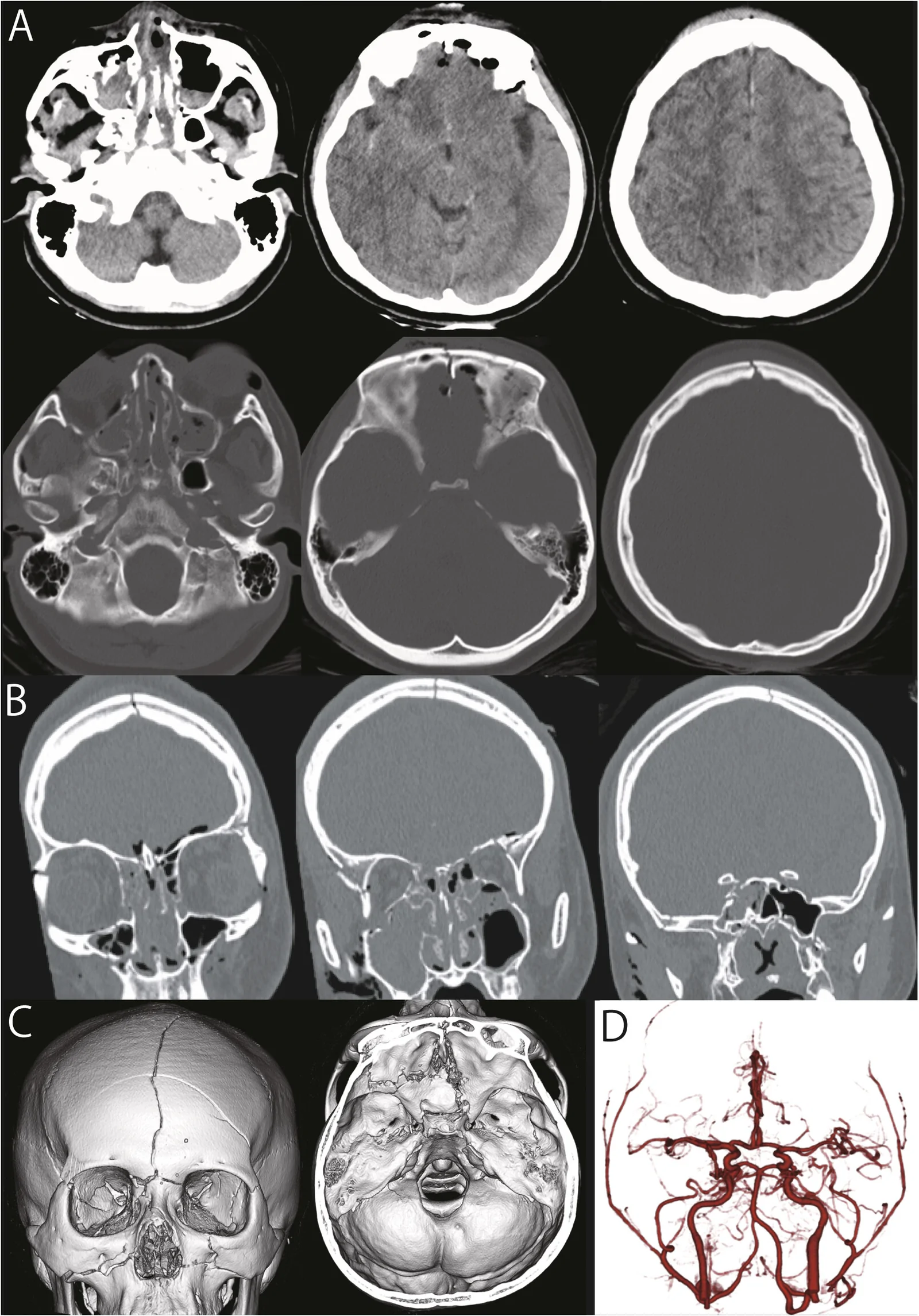
Initial radiological findings upon admission. ( **A** ) Axial head computed tomography (CT) and ( **B** ) coronal head CT. ( **C** ) Front and top views of three-dimensional (3D) reconstruction demonstrating anterior skull base fractures. ( **D** ) 3D CT angiography showing no evidence of vascular injury.

Fiberoptic examination on day 2 revealed pulsatile CSF leakage. Despite lumbar drainage being initiated on day 4, CSF rhinorrhea worsened, and surgical repair was performed on day 7.

## Operative Technique

A 4-mm 0-degree 4 K endoscope (Karl Storz) was used.

A bicoronal incision was made, and an approximately 15-cm pericranial flap was created ( Fig. 2A ). The skin flap was dissected using an interfascial technique to preserve the facial nerve. Medial dissection was performed until the supraorbital nerves were visible. Longitudinal and transverse fracture lines were identified across the frontal midline ( Fig. 2B,C ). We avoided a longitudinal fracture line and used a transverse fracture line instead to minimize the risk of venous sinus injury. Bilateral MacCarty keyholes were drilled, followed by bilateral supraorbital craniotomies measuring approximately 5 cm and extending medially ( Fig. 2D,E ). No dural tears occurred during craniotomy.

**Fig. 2:**
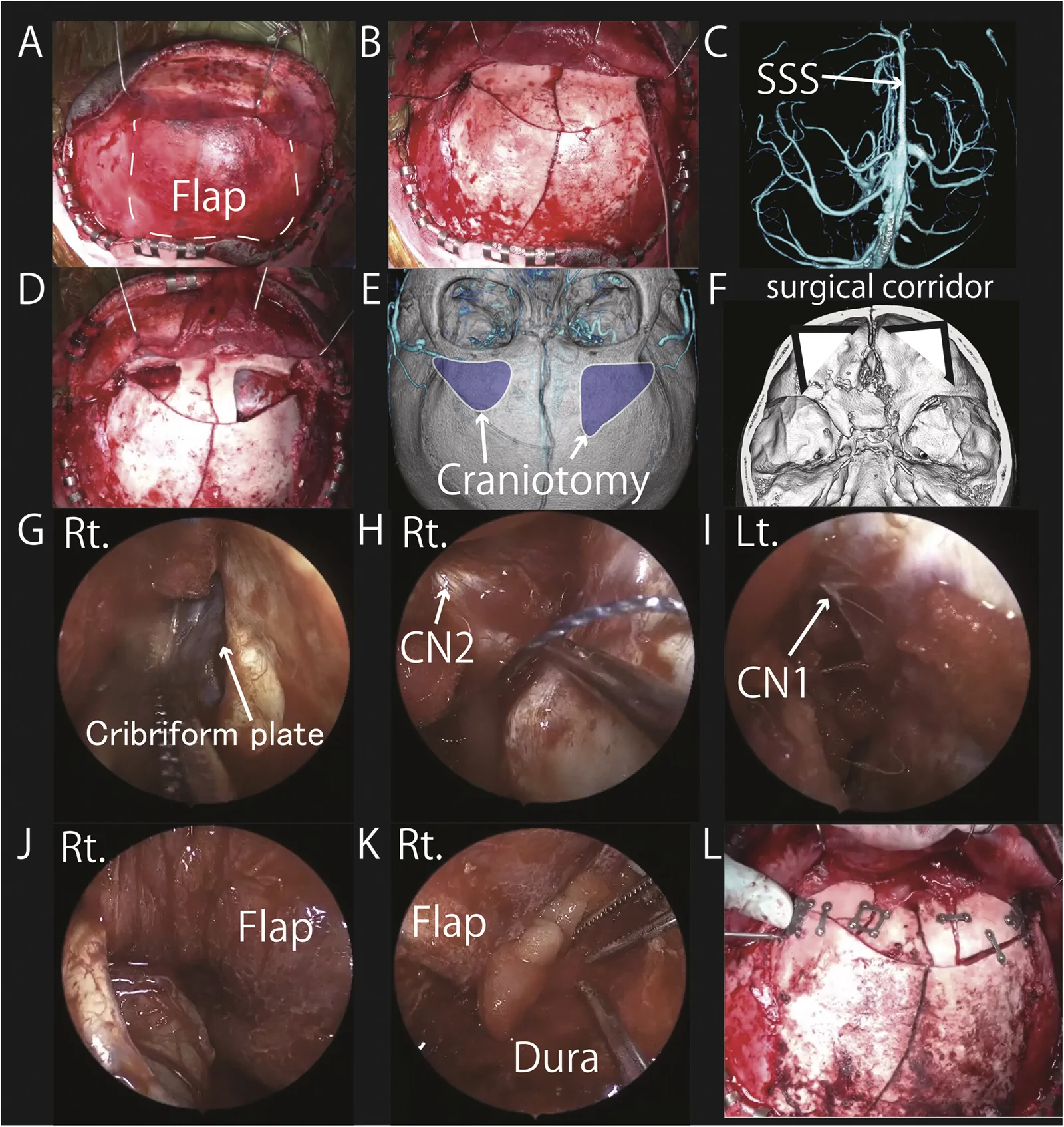
Intraoperative photographs and 3D CT reconstructions demonstrating the surgical sequence. ( **A** ) Bicoronal skin incision and harvested pericranial flap. ( **B** ) Visualized frontal bone fractures following skin flap dissection and ( **C** ) 3D CT angiography showing the superior sagittal sinus. ( **D** ) Bilateral supraorbital craniotomies and ( **F** ) visualization of the surgical corridor on 3D skull base reconstruction. ( **G** – **K** ) Intraoperative endoscopic views demonstrating ( **G** ) herniation of brain parenchyma into the skull base, ( **H** ) severe damage to the right optic canal, and ( **I** ) preserved left olfactory nerve. ( **J** ) Placement of the split pericranial flap. ( **K** ) Completion of multilayered dural closure. ( **L** ) Final operative view after fixation of the bone flaps. Abbreviations: CN1, olfactory nerve; CN2, optic nerve; Dura, dural matrix; Flap, pericranial flap; Lt., left side; Rt., right side; SSS, superior sagittal sinus.

This approach was initially performed on the right side. After making a dural incision approximately 2 cm parallel to the anterior skull base, an endoscope was inserted ( Fig. 2F ). Endoscopic inspection revealed severe herniation of the brain parenchyma through the skull base, near the cribriform plate ( Fig. 2G ). This herniation point was suspected to be the primary site of the CSF leakage. The right olfactory nerve could not be visualized and was presumed to be disrupted. Extensive damage was also observed around the right optic canal ( Fig. 2H ), suggesting associated optic nerve injury.

The dura was subsequently opened on the left side. The left olfactory nerve appeared to be morphologically preserved ( Fig. 2I ). As discrete CSF leakage could not be identified, a broad reconstruction of the entire anterior skull base was performed. The pericranial flap was divided at the midline and placed on the anterior skull base covering the cribriform plate under endoscopic guidance ( Fig. 2J ). An absorbable collagen dural matrix (DuraGen; Integra Life Sciences Corp) was used to achieve multilayered reconstruction, and a fibrin sealant was applied. The pericranial flap was then sutured securely to the incisional edges of the native dura to achieve a watertight closure ( Fig. 2K ). Finally, the bone flaps were fixed ( Fig. 2L ), and the wound was closed in layers. The operative time was 4 hours 25 minutes, and the estimated blood loss was 180 mL.

## Results

No postoperative recurrence of CSF leakage was observed, and the lumbar drain was successfully removed. Postoperatively, she exhibited left oculomotor nerve dysfunction but was able to communicate without difficulty and did not complain of any ocular symptoms. She subsequently underwent surgery for the limb and facial fractures.

Follow-up 3D CT angiography performed 14 days after the injury ( Fig. 3A ) showed a weak contrast-enhancing effect in the cavernous sinus without swelling of the cortical veins. Since she did not complain of double vision or tinnitus, we carefully monitored her progress using short-term follow-up imaging. Although no delayed vascular injury was demonstrated by repeat 3D CT angiography performed 3 months after the injury ( Fig. 3B ), she developed a visual field defect approximately 6 months’ postinjury with no worsening of her ocular motility disorders. There were no other ocular symptoms such as redness or pulsation. Orbital examination revealed a corrected counting finger visual acuity of 50 cm in the right eye and 0.2 in the left eye. Bitemporal hemianopsia was suspected based on confrontation visual field testing; however, no clear findings were observed in the anterior or posterior segments of the eye. Further vascular evaluation is required to rule out delayed traumatic arteriovenous shunt disease. Due to the presence of numerous metal implants that precluded the use of magnetic resonance imaging, a common carotid artery angiography was performed ( Fig. 3C ). Angiography revealed no vascular lesions, and the patient was managed with continued observation for neurological symptoms. Although her objective visual function, including field of vision and visual acuity, did not fully recover, she regained independence in daily activities and was discharged 8 months after the injury. The patient’s long-term neurological outcomes remained stable at 3-year follow-up. Despite her visual deficits, her remaining eyesight was sufficient for her to perform activities of daily living. No delayed surgical complications were observed.

**Fig. 3:**
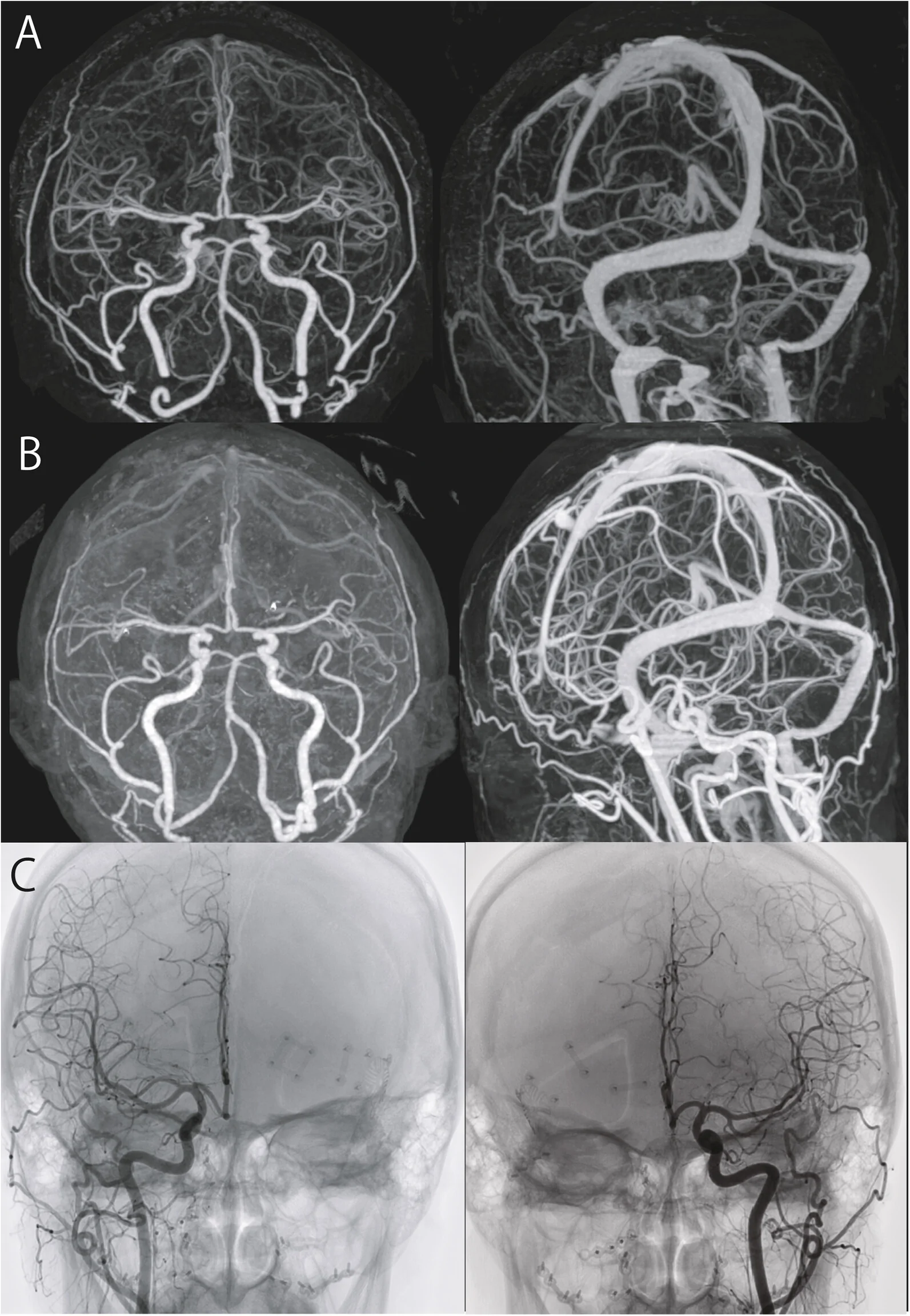
Postoperative imaging. ( **A** ) Three-dimensional (3D) computed tomography (CT) angiography at 14 days’ postinjury showing a weak contrast-enhancing effect in the right cavernous sinus. No vascular abnormalities were detected on subsequent imaging. ( **B** ) 3D CT angiography at 3 months’ postinjury. ( **C** ) Common carotid artery angiography at 6 months’ postinjury.

## Discussion

Conservative management of traumatic CSF leaks includes head elevation, avoidance of straining, and lumbar drainage. 4 6 However, prolonged management beyond 1 week has been associated with a significant increase in meningitis risk from 5–11% to 55–88%. 1 Therefore, surgical intervention is strongly recommended for persistent or delayed CSF leakage. 1 4

Historically, surgeons have selected traditional craniotomy or endoscopic endonasal repair. Endoscopic surgery has demonstrated excellent outcomes for well-selected anterior skull base fractures, including lower failure rates (2.8% vs. 5.3%) and fewer postoperative complications (0.7% vs. 11.1%) compared with traditional craniotomies. 7 8 Nevertheless, purely endoscopic approaches may be insufficient in cases involving large dural defects, extensive bone fragmentation, or uncontrolled hemorrhage, in which craniotomy approaches remain indispensable. 3 9

Regardless of the approach, watertight multilayered closure is the cornerstone of successful CSF leak repair, because continuous CSF pulsation and hydrostatic pressure are major causes of repair failure. 10 11 12 13 Combining autologous vascularized pedicled flaps with modern artificial dural substitutes has substantially improved endoscopic skull base reconstruction outcomes. 11 12 These robust reconstruction principles are also highly valuable for complex trauma cases.

Advances in endoscopic technology have also transformed skull base surgeries. Enhanced visualization now enables smaller, more targeted craniotomies without compromising repair quality. The supraorbital subfrontal keyhole approach is widely adopted because it reduces approach-related morbidity. 14 15 In the present case, conservative management with lumbar drainage failed to resolve the CSF leak and required surgical intervention. Endonasal repair was considered; however, the extent of the bilateral fractures spanning from the frontal bone to the optic canals and the difficulty in localizing the exact leak point made a purely endonasal approach less suitable. Repairing massive anterior skull base defects via the endonasal route is challenging. Specifically, the anterior reach and surface area of a nasoseptal flap via an endonasal approach are often insufficient to adequately cover extensive anterior skull base defects such as the one in this case. 10 Moreover, endoscopic approaches have anatomical limitations regarding visualization and instrumentation when accessing widespread fractures involving the lateral frontal sinus and orbital roof. 10 Furthermore, lateral extensions of frontal sinus fractures are considered contraindications for an endoscopic approach because the endonasal route is suitable for precisely located small tears. 3 In addition, gaining wider endoscopic access would necessitate additional bone drilling. Removing more bone from an extensively fractured skull base increases surgical morbidity and the risk of a recurrent CSF leak. Therefore, a transcranial approach is preferred to provide the necessary wide exposure and secure reconstruction.

Although supraorbital approaches, endoscopic assistance, and pericranial flap reconstructions have been established individually, the principal novelty of our approach is their synergistic combination for complex skull base trauma. Traditionally, achieving deep and robust flap coverage for widespread fractures requires a large bifrontal craniotomy, which is associated with significant morbidities, notably vascular injury and postoperative epidural fluid collection secondary to massive dural detachment. The proposed technique circumvents these issues. By utilizing tailored bone windows that deliberately bypass the unstable fracture lines and critical anatomy, unnecessary dural peeling is minimized. Concurrently, the endoscope compensates for the limited physical corridors by providing the deep, wide visualization necessary to securely place the pericranial flap without recurrence. Ultimately, this combination enables definitive reconstruction using a large-open approach.

This strategy may be particularly well suited for acute trauma settings. Emergency extensive craniotomies pose a significant risk of surgical site infection owing to the prolonged operative time and extensive tissue exposure. 16 17 In contrast, our tailored approach may mitigate these risks by reducing the surgical duration, avoiding venous sinus exposure, and minimizing the manipulation of fragile tissues. Therefore, this technique represents a safe and highly effective alternative for patients with complex fractures.

## Limitations

This study had some limitations. First, it describes a single case, which limits generalizability. Second, the exact CSF leakage could not be identified. Because the patient presented with severe polytrauma requiring semi-emergency surgery, additional localization studies—such as CT/magnetic resonance cisternography or intrathecal fluorescein—were clinically impractical. Therefore, a broad reconstruction strategy was adopted to ensure complete coverage and may have potentially overestimated the required repair area. However, further studies are required to establish the validity of this approach.

## Conclusions

We report a case of traumatic CSF rhinorrhea caused by an anterior skull base fracture that was successfully treated with endoscope-assisted repair through tailored bilateral supraorbital craniotomies. By deliberately avoiding unstable fracture lines, the risks of postsurgical infection and venous sinus injury were minimized. Endoscopic visualization compensated for the restricted operative field. Endoscopic CSF leak closure via minimal craniotomy is an effective and viable surgical approach for select cases of extensive anterior skull base fractures.

## Declaration of GenAI Use

The authors used Google Gemini (Gemini 3 Flash) to assist with linguistic refinement and grammatical corrections. We reviewed and verified all AI-generated suggestions to ensure scientific accuracy.
